# How I Treat: Transplantation for primary immune regulatory diseases and inflammatory disorders

**DOI:** 10.70962/jhi.20250147

**Published:** 2026-10-05

**Authors:** Joseph Hai Oved

**Affiliations:** 1 Section of Transplantation & Cellular Therapies, Departments of Pediatrics & Medicine, Columbia University Irving Medical Center, New York, NY, USA

## Abstract

Primary immune regulatory disorders (PIRDs), autoinflammatory, and hyperinflammatory syndromes can be life-threatening and/or significantly impact quality of life. Targeted immune modulation may control symptoms for some, but allogeneic hematopoietic stem cell transplantation (alloHCT) remains the only cure for many patients. Despite advances in alloHCT, outcomes for this population continue to need optimization. Focus areas include referral timing, patient selection, multidisciplinary evaluation, targeted pretransplant immune suppression (PTIS), graft selection, model- and exposure-based conditioning, and post-alloHCT management. Here, we describe our preferred alloHCT approach for this patient population. This includes early alloHCT referral, continued evaluation to identify an appropriate alloHCT window, 6–12 wk of targeted PTIS for inflamed patients, a myeloablative model-based reduced-toxicity conditioning regimen, a strong index of suspicion for posttransplant immune dysregulation, and multidisciplinary long-term follow-up. Emphasis is placed on the need for predictive biomarkers to guide clinical decision-making, objective measurements of long-term response/disease resolution, and prospective multicenter trials. By refining our approaches, curative outcomes will continue to improve.

## Teaser

Primary immune regulatory disorders (PIRDs), autoinflammatory, and hyperinflammatory syndromes can be life-threatening and/or significantly impact quality of life. Targeted immune modulation may control symptoms for some, but alloHCT remains the only cure for many patients. Despite advances in alloHCT, outcomes for this population continue to need optimization.

## Introduction

The primary immune regulatory disorders (PIRDs), autoinflammatory, and hyperinflammatory syndromes encompass a rapidly growing constellation of diseases with overlapping clinical phenotypes and molecular findings (1, 2). While primary immune deficiencies are characterized by severe opportunistic infections, these disorders manifest with immune dysregulation, hyperinflammation, autoimmunity, lymphoproliferation, malignancy, opportunistic infections, and atopy (3). This spectrum of disease can be characterized by primarily autoimmune sequelae (e.g., IPEX syndrome), those that manifest with mostly hyperinflammation (e.g., primary hemophagocytic lymphohistiocytosis [pHLH] syndromes), and those that are a mix of both autoimmune complications and dysregulated inflammation (e.g., STAT1 gain-of-function [GOF]). Given the overlapping phenotypes and sometimes mild initial presenting symptoms, diagnosis can be difficult and can even take years, involving a combination of functional and genetic testing to isolate a causative defect (4).

Despite the sometimes-mild presentation and insidious progression of disease, the chronic underlying immune dysregulation and hyperinflammation can cause significant end-organ damage over time. One of the major target organs where this is most apparent is the lungs. For instance, patients with JAK/STAT dysregulation, LRBA deficiency, CTLA4 haploinsufficiency, systemic juvenile idiopathic arthritis (sJIA), and other disorders can have a component of inflammatory lung disease that leads to fibrosis over time (5, 6). Frequent, chronic, and superimposed upper respiratory infections can cause additional significant lung injury and lead to the development of bronchiectasis (7). Though symptoms may be modest at first, the progressive nature of bronchiectasis poses a tremendous burden on quality of life for affected patients and is often a major factor in disease-related morbidity and mortality (8).

The introduction of targeted therapeutics has changed the treatment landscape of patients with PIRDs and inflammatory disorders. Drugs such as abatacept for LRBA deficiency and CTLA-4 haploinsufficiency (9, 10), JAK inhibitors (JAKinibs) for JAK/STAT GOFs (11, 12), anifrolumab for type 1 interferonopathies (13, 14) and emapalumab for type 2 interferonopathies, IFN-γ hyperinflammation, and HLH (15, 16, 17, 18) enable disease control by modulating the dysregulated pathways involved in clinical pathology. The ability to control dysregulated immunity and inflammation with these targeted drugs promotes organ recovery and some tissue remodeling in patients (12). Though these drugs are not curative, a cohort of patients do very well on targeted therapeutics over the long term (19). Others, however, only have partial response or breakthrough over time and require additional interventions. While alternative therapeutics may be trialed, allogeneic hematopoietic stem cell transplantation (alloHCT) remains the only available curative approach for many of these disorders (11, 20).

AlloHCT has been used over the past several decades to attempt to cure patients with PIRDs and inflammatory disorders (20). Historically, alloHCT was reserved for certain conditions, for patients with an available, unaffected HLA-matched related donor (MRD) and/or matched unrelated donor (MUD), or for patients without any other available therapeutic options (20). Outcomes for patients treated with alloHCT were not optimal, with the largest multi-institutional series of alloHCT for PIRD and/or autoinflammation reporting a 5-year overall survival (OS) of 67% for 226 patients treated at 33 centers in the United States and Europe from 1982 to 2017 (20). Notably, 75% of patients in this cohort received an MRD/MUD graft, and disorders such as STAT1 GOF and other type 2 interferonopathies were relatively underrepresented (20). In a separate report on the initial multicenter experience of alloHCT for 15 patients with STAT1 GOF, event-free survival (EFS) was only 10% at day +100, and OS at the time of data collection was only 40% (21). Thus, while alloHCT represents the best opportunity for cure, optimization of patient selection, pre-HCT interventions, and HCT procedures are required to provide benefit to the patients that need it most.

Modern advances in alternative donor HCT, supportive care, and conditioning regimens have improved alloHCT outcomes across all diseases and have led to consideration of new indications for curative therapy (22). When considering potential inborn errors of immunity (IEIs) as candidates for cure, it is critical to define syndromes such as thymic education defects, APECED, NFKB2, and complement defects (with the exception of C1q defects) that would not improve with alloHCT. Outcomes for patients with PIRDs, autoinflammation, and/or hyperinflammation continue to lag behind the outcomes of other nonmalignant alloHCT indications such as severe acquired aplastic anemia, severe combined immunodeficiency (SCID), Wiskott–Aldrich syndrome, and chronic granulomatous disease, which have an OS >85% and approaching >90% in many cohorts (23, 24, 25, 26, 27, 28, 29). Emerging data on the negative effects of inflammation, and more specifically IFNγ and related pathways on hematopoiesis and graft function may partially explain the difference in EFS and OS for patients with PIRDs and/or inflammatory disorders (30, 31, 32, 33). The exact role of IFNγ and graft dysfunction/rejection is still being studied, but data in murine models and humans have shown that elevated CXCL9, a validated biomarker of activated IFNγ (16, 34), is associated with graft rejection (30, 31, 35). In fact, in the TCRαβ depletion strategy of haploidentical grafts, an elevated CXCL9 at day +3 was predictive of graft rejection (31). IFNγ and complement pathway dysregulation have also been implicated in increased endothelial damage and the sequelae thereof (36). Thus, patients with PIRDs, autoinflammation, or hyperinflammation that have constitutive inflammation and IFNγ activation are at a disadvantage prior to even beginning conditioning for alloHCT.

Due to the negative effects of inflammation on alloHCT and graft function, various bridging strategies have been employed to attempt to quell inflammation leading into conditioning and alloHCT (11, 37, 38, 39, 40). In the most recent cohort of patients with STAT1 GOF that underwent alloHCT, those that received pretransplant immune suppression (PTIS) with a JAKinib had an EFS and OS of 73% and 84% compared with an EFS and OS of 30% and 54% in patients that did not receive a JAKinib bridge to alloHCT (41). A similar strategy using emapalumab as a bridge has also been successful in STAT1 GOF (42) and is currently in clinical trial (NCT05787574) for patients with PIRDs undergoing alloHCT. Emapalumab has also been used to bridge patients with pHLH to alloHCT, with improved donor engraftment in patients who received a reduced-intensity regimen (43). Similarly, abatacept has been used to bridge patients with LRBA deficiency or CTLA4 haploinsufficiency to alloHCT (38). Other more general strategies of PTIS have also been employed in IEI as well as other nonmalignant diseases with a high degree of marrow inflammation (e.g., hemoglobinopathies) (44, 45, 46). The benefits of PTIS and bridging to alloHCT likely extend beyond starting alloHCT from a non-inflamed baseline, and allow for some tissue remodeling and organ improvement depending on the length of PTIS. This strategy is therefore a major advance in providing cures with alloHCT for patients with PIRDs and/or inflammatory disorders.

## Who and when?

**Figure 1.**
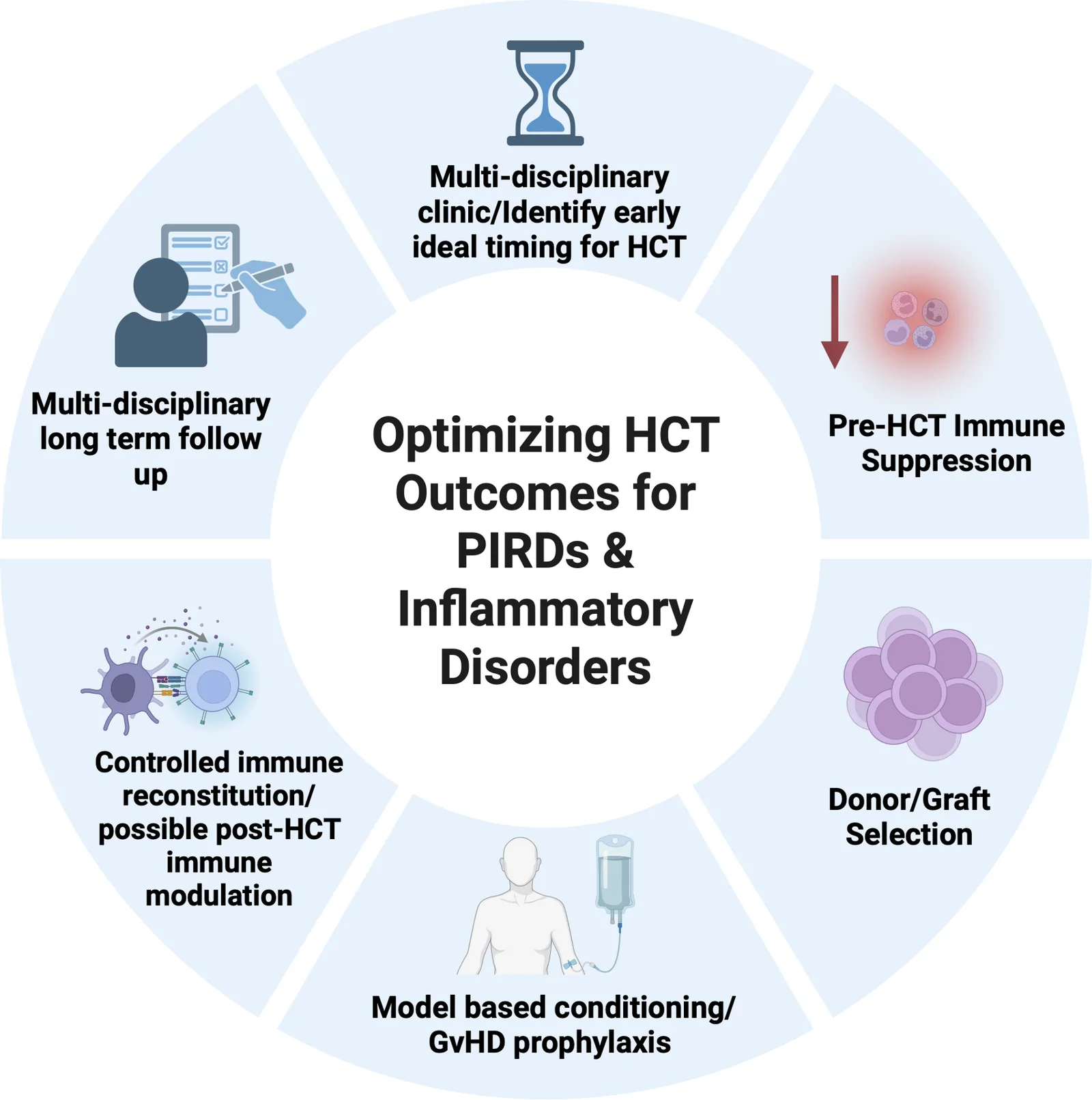
**Optimizing HCT outcomes for PIRDs and inflammatory disorders.** The various factors before and after HCT that can be modified to optimize outcomes include a multidisciplinary approach to identify ideal timing to pursue curative therapy, consideration of targeted pre-HCT immune suppression as a bridge to HCT, optimal donor and graft selection, model-based conditioning with tight control of exposures, deep GVHD prophylaxis, controlled immune reconstitution with constant consideration of post-HCT immune modulation as needed, and long-term multidisciplinary follow-up.

The most difficult dilemma for patients and providers when considering alloHCT for these disorders is who would benefit from this approach and when is the ideal time to proceed (Fig. 1) (47). This is especially true given that many of the genetic alterations have incomplete penetrance, skewed monoallelic expression, and can have vastly different clinical phenotypes even within the same family (48). The absence of robust predictive biomarkers makes prognosticating even more difficult. Lack of functional validation of many genetic variants and rare cases of somatic reversion complicate matters further (49). In a small number of HLH-associated genes, preemptive alloHCT has been effective, but the availability of these datasets is the exception and not the norm (50, 51). Current efforts of screening newborns with whole-genome sequencing have made the issue of when to pursue alloHCT more acute (52). For example, several recent cases from genomics-based screening that were referred for an initial consultation include a patient with a FOXP3 δ exon 2 (53) and an additional patient with an IL6-ST dominant negative variant and a second IL6-ST variant of unknown significance, which both generated robust discussions among our team with an ultimate decision to monitor closely for any clinical sequelae of disease, but not pursue alloHCT at this time.

Given the complexities of these disorders, the many unknowns, and the rapidly evolving landscape, a multidisciplinary approach is necessary for optimal care (39). While several centers have created a multidisciplinary HLH rapid response team, these teams are less frequently available for PIRDs. The dysregulated immune response team at Children’s Hospital of Philadelphia is a model of a comprehensive multidisciplinary approach to patients with PIRDs, autoinflammation, and hyperinflammation, though one drawback is that it lacks the longitudinal emphasis to evaluate adult patients with these disorders. This is critical, as more patients with these underlying disorders are living to adulthood and need continued evaluation while adult providers do not necessarily have the same experience and expertise treating these patients (54, 55, 56). For centers that lack the ability to form a multidisciplinary program, national consortium boards such as the Primary Immune Deficiency Treatment Consortium (PIDTC) IEIs board provide an opportunity to solicit consensus expert opinion and can be invaluable in helping to adjudicate potential next steps for complex patients.

Our approach at Columbia University Irving Medical Center Program for Immune Dysregulation has evolved and favors early alloHCT. The reason for this is that evidence shows that patients accumulate end-organ toxicities over time, even if they are not clinically apparent. The goal for alloHCT is to cure the intrinsic hematopoietic defect while maintaining as much organ function and quality of life as possible. Since most organ damage cannot be reversed by alloHCT, we prefer to intervene early, while the additional risk of end-organ damage going into alloHCT is minimal. We try to balance this approach of early intervention, recognizing the added toxicity of cytoreduction medications such as busulfan and other alkylating agents on younger children while also giving time for a specific mutation to clinically “declare” itself (57, 58).

### Case 1

A baby was born at 32-wk gestation with a pathogenic IKBKG mutation identified based on family history and consistent with NEMO deficiency. This specific mutation was particularly pathogenic in the affected family, with most affected males having died by age 8 years. The patient was referred for preemptive alloHCT and discussed extensively in our multidisciplinary group. We agreed that alloHCT with the best available donor (a MUD in this case) could be appropriate given prior family history but preferred to delay as long as possible to give the patient time to grow. We recommended starting the patient on an antimicrobial and anti-mycobacterial prophylaxis regimen as well as regular intravenous Ig (IVIG) supplementation. We also instituted monthly assessments of inflammation, including calprotectin to assess for any gastrointestinal (GI) inflammation, with the plan of intervening as soon as markers of inflammation started to increase. After several months, the patient started to develop some early laboratory signs concerning for inflammation as well as early lymphedema, similar to other affected family members, and we reassessed and decided in close consultation with the family and primary providers to move forward with alloHCT. Thus, while we want to intervene early for many patients, we find that for some, waiting with very close monitoring and prophylaxis is appropriate and sometimes beneficial. It also provides an opportunity for a mutation to prove its clinical penetrance in an individual patient. The potential downside to this approach is the need to act quickly if clinical signs emerge, and the inability to predict if the first clinical flare will be severe.

## Pre-HCT optimization

A major predictor of alloHCT outcomes is the condition in which patients are in as they begin the process. This is captured by the HCT comorbidity index (HCT-CI) score, which includes 17 clinical parameters (59, 60). A higher HCT-CI score indicates more significant pre-alloHCT comorbidities and correlates with poorer post-alloHCT outcomes (61). A pediatric version of the HCT-CI score has also been validated for nonmalignant alloHCT (62, 63). While the HCT-CI score is very useful in accounting for certain organ toxicities in assessing risk of alloHCT, it does not account for immune dysregulation or hyperinflammation. To assess the state of immune dysregulation, other scoring systems must be used (64). The Immune Deficiency and Dysregulation Activity (IDDA2.1) score consists of 22 parameters to comprehensively evaluate the level of immune dysregulation (65, 66). When both scores were used to determine patient risk, patients with high HCT-CI scores (>3) and high IDDA2.1 scores (>15) unsurprisingly had inferior alloHCT outcomes with a 3-year OS and EFS of 70% and 30% compared with an OS and EFS of 100% and 93% in patients with low HCT-CI (<3) and IDDA2.1 scores (<15) (67). Notably, patients with a low HCT-CI (<3) and a high IDDA2.1 score (>15) who would have been deemed low risk by HCT-CI standards had OS and EFS of 96% and 76%, and therefore their EFS risk profile would have been underestimated without the IDDA2.1 score (67). Thus, for patients with PIRDs and/or inflammatory disorders, immune dysregulation, as measured by the IDDA2.1 score, represents an additional modifiable risk factor that must be accounted for when considering curative therapy with alloHCT (67). Other scoring systems such as the PIDTC immune monitoring and phenotype assessment of clinical trajectory score with 15 modules assessing 196 disease conditions are currently being validated as well (68).

The increased risk posed by uncontrolled immune dysregulation on alloHCT outcomes, as well as the recent advances in targeted immune modulators, have led to the concept of bridging to alloHCT with targeted immune modulation (37, 41). Our approach to bridging with PTIS relies on determining an IDDA2.1 score at referral. If the IDDA2.1 score is >15 or there is significant inflammatory lung disease (or other inflammatory organ disease), we attempt to modulate the immune dysregulation with targeted PTIS. We generally attempt a 6-wk trial of the targeted immune modulation and then reassess with appropriate diagnostics and a new IDDA2.1 score. If there is improvement, we will start working toward alloHCT with the goal of having a 6–12-wk total length of bridging PTIS before the preparative regimen.

### Case 2, part 1

A patient with an *IKZF1* GOF was referred for alloHCT. He had significant inflammatory lung disease and, despite being on immune suppression, had lymphoproliferation and splenomegaly that would have made engraftment difficult. A 6-wk trial of ruxolitinib was initiated with improvement in inflammatory lung disease, splenomegaly, lymphoproliferation, and IDDA2.1 score. Ruxolitinib was continued for an additional 6 wk for further improvement, during which time we began preparing for alloHCT.

A critical factor in bridging immunomodulatory therapy is when and how to discontinue leading into alloHCT. Our preferred approach is to taper therapy over several weeks and into the serotherapy portion of the conditioning regimen. This is particularly important for JAKinibs, because JAKinibs withdrawal can develop if treatment is not tapered appropriately (69, 70). In general, we aim to have PTIS off by the start of serotherapy or chemotherapy depending on the half-life of the targeted therapeutic. For patients in which tissue resident immune cells may continue to cause significant dysregulation and/or for some autoinflammatory disorders, we may continue immune modulation through engraftment (and longer). If posttransplant cyclophosphamide (PTCy) is being used as graft-versus-host disease (GVHD) prophylaxis, the benefit of additional immune modulation must be balanced with the potential for decreasing the efficacy of the PTCy. Thus, a cautious, personalized, and mechanistic-based approach is critical to tailoring or continuing PTIS. If CXCL9 or soluble IL2 receptor is elevated as PTIS is tapered, we consider adding emapalumab 3 mg/kg to our conditioning regimen (described below). The benefits of emapalumab are that it neutralizes IFNγ, which has been implicated in allograft rejection as well as sequelae of alloHCT caused by endothelial damage (35, 36, 71). Unlike some JAKinibs, emapalumab does not cause cytopenias, which is of particular importance during the peri-engraftment period. It also has a relatively long half-life (∼23 days and inversely proportional to CXCL9 levels) (72) and therefore provides a self-tapering targeted immune suppression in the first months after alloHCT (42).

One particular concern that has been seen with bridging PTIS is early viral reactivation. This has occurred with CMV, EBV, and BK virus and is likely not specific to these three viruses (73, 74, 75). Though often low-level reactivation occurs, CMV in particular may require treatment in the first few days after allograft infusion. We therefore have instituted an aggressive antiviral strategy with the start of bridging PTIS. We evaluate CMV, EBV, adenovirus, and BK virus PCRs at the initiation of PTIS. If viral PCRs are negative, we institute weekly viral PCR monitoring for CMV, EBV, and adenovirus. If a patient is CMV seropositive and PCR negative, we either start letermovir or valganciclovir/ganciclovir (maintenance dosing) immediately. If the CMV PCR becomes positive, we increase from maintenance to treatment dosing with the goal of achieving a negative PCR by the start of conditioning. If ganciclovir is used, we transition to letermovir on day −1. If there is persistent and/or rising EBV reactivation, we consider adding a single dose of rituximab, prior to start of conditioning to prevent post-alloHCT complications. For patients receiving a TCRαβ T cell–depleted graft, rituximab is often incorporated into standard conditioning regimens to prevent posttransplant EBV complications. To date, we have not seen clinically significant adenovirus reactivation during PTIS, but we would approach similar to the posttransplant patient with either cidofovir and/or virus-specific cytolytic T cells (VSTs). A phase 3 international trial of i.v. brincidofovir for adenovirus reactivation is currently accruing for posttransplant patients (NCT07387367). For BK virus, we recheck PCR at the end of PTIS and prior to conditioning, though even if there is reactivation, we have not intervened. Using this approach, we have been able to adequately treat and avoid serious sequelae of early viral reactivation. While we have an internal VST program that we sparingly use for this patient population, it is important that centers doing alloHCT for these conditions have access to a robust internal or external VST program if necessary.

Antibody-mediated pathologies (e.g., immune-mediated cytopenias) are also a concern that need to be addressed prior to alloHCT. In general, agents such as rituximab, bortezomib, and daratumumab are helpful to acutely mitigate pathology. We try to use these therapeutics sufficiently prior to alloHCT so that there is minimal residual circulating drug, as the effects on B cell reconstitution (rituximab) and engraftment (daratumumab) have not been adequately studied (76, 77). If patients recur prior to alloHCT, we attempt to recapture with these agents and then use medications such as mycophenolate mofetil (MMF), sirolimus, etc. to maintain adequate cell counts prior to starting conditioning. It is critical to optimize immune-mediated cytopenias going into alloHCT, as these antibodies can negatively affect engraftment kinetics. It is also critical to assess for donor-specific antibodies in this patient population and to follow institutional protocols if they are present.

## Donor selection and graft source

**Figure 2.**
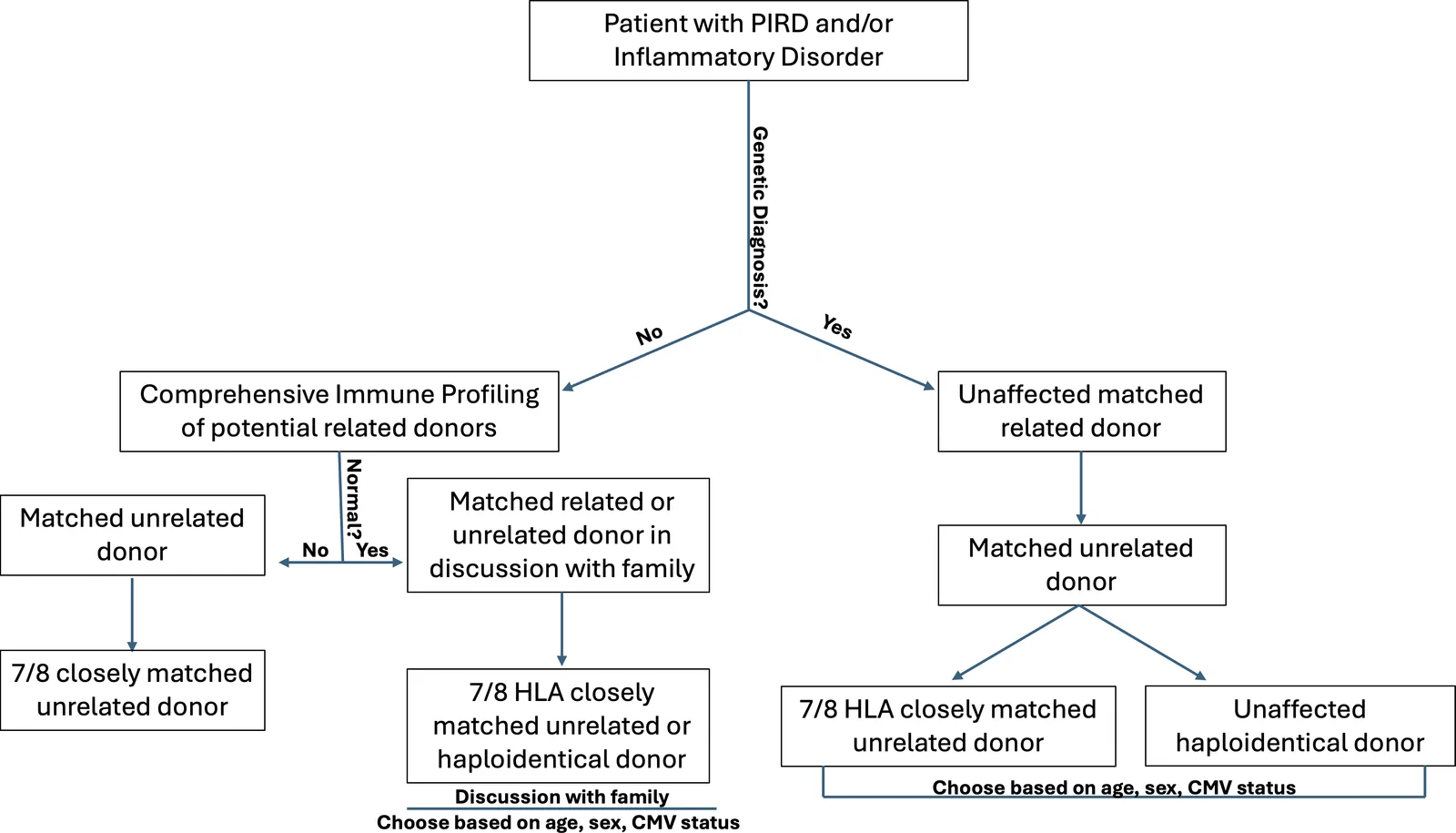
**Donor selection workflow.** For patients with a genetic diagnosis, we prefer an unaffected MRD followed by a MUD and would consider a 7/8 HLA closely MUD or an unaffected haploidentical related donor depending on age, sex, and CMV status. In patients who do not have an identified genetic etiology, we pursue comprehensive immune profiling of potential related donors and, based on these data, either choose the best available unrelated donor or have discussions with the family to promote shared decision-making in choosing the best available related versus unrelated donor.

As in all instances of alloHCT, an available unaffected matched sibling donor (MSD) is the gold standard (75). The caveat for patients with PIRDs and/or inflammatory disorders is what to do if an underlying genetic etiology is not identified in the patient. In these cases, we typically would prefer to use a MUD if one is available, particularly if the matched sibling is younger than the patient when they first presented with clinical symptoms (Fig. 2). This has come up several times in patients with sJIA with no identified underlying genetic etiology that have an available MSD. We have sometimes pursued comprehensive immune profiling of the sibling. If immune profiling is completely normal, we have a discussion with the family about the risks and benefits of using the sibling versus a MUD with the goal of promoting a shared decision-making process. Similarly, in patients who do not have an identified genetic etiology and also do not have a MUD, we would prioritize a 7/8 closely MUD over a haploidentical donor, although this is a physician- and institution-specific prioritization. For patients with an identified genetic lesion, our prioritization would be available unaffected MSD followed by MUD followed by either a 7/8 closely MUD or a haploidentical donor depending on donor age, sex, and CMV status. If the haploidentical and the closely MUD were deemed equal, we would prioritize the haploidentical donor, particularly if we wanted to use a bone marrow graft. Cord blood represents another excellent graft source that should be considered if an appropriate unit is available (78). The nuances involved in unit selection are beyond the scope of this paper; however, cord blood units should only be used at centers that have expertise in this approach. The National Cord Blood Network has been established as a resource for centers that are considering a cord blood transplant and can assist with unit selection, training, and standard operating protocols.

The question of bone marrow allografts or mobilized peripheral stem cells depends on whether ex vivo graft manipulation or in vivo T cell depletion is being utilized. As described below, we prioritize an in vivo T cell depletion approach and, as such, prefer bone marrow allografts when possible to minimize the potential for GVHD (79, 80). The major caveat is making sure there is an adequate CD34^+^ cell dose. In a phase 2 multicenter study of alloHCT for severe acquired aplastic anemia, a bone marrow allograft with a total nucleated cell (TNC) count <2.5 × 10^8^ cells/kg was associated with graft rejection (81). Extrapolating from this experience, we target a TNC of 3–5 × 10^8^/kg and/or CD34^+^ count of 3–5 × 10^6^ cells/kg (similar to EBMT guidelines) with a cap of 10 × 10^6^ CD34^+^ cells/kg to avoid increased risk of GVHD as well as potential risk of increased inflammation if there is too robust an engraftment (82). If we are ever doubtful that we will obtain at least 2 × 10^6^ CD34^+^ cells/kg, we select a mobilized peripheral stem cell graft and cap our CD34^+^ cell dose at 7.5–8.0 × 10^6^ cells/kg (82). We modulate this number based on CD3^+^ cell counts to balance GVHD risks. For patients receiving ex vivo T cell–depleted grafts, mobilized peripheral stem cell grafts are preferred, and high CD34^+^ cell doses are often utilized.

## Conditioning regimen

The conditioning regimen for patients with PIRDs, autoinflammation, or hyperinflammation consists of three components—serotherapy, myeloablative agent(s), and immune ablative agent(s). For serotherapy, there are two main therapeutics: alemtuzumab, a humanized anti-CD52 monoclonal antibody, and rabbit antithymocyte globulin (rATG), a polyclonal rabbit-derived antibody against human thymocytes (83). Both alemtuzumab and rATG have their pros and cons, and both are excellent immune-depleting agents. Our focus has been on rATG (specifically thymoglobulin) due to the extensive population pharmacokinetic (PK) modeling and immune reconstitution studies that have previously been completed (84, 85, 86, 87, 88). While PK models exist for alemtuzumab as well, tightly controlled exposure-based dosing is still being refined (89, 90, 91). We therefore incorporate exposure-based rATG dosing using the population-PK model from the PARACHUTE trial, which emphasizes high pre-HCT and low post-HCT exposure (87).

**Figure 3.**
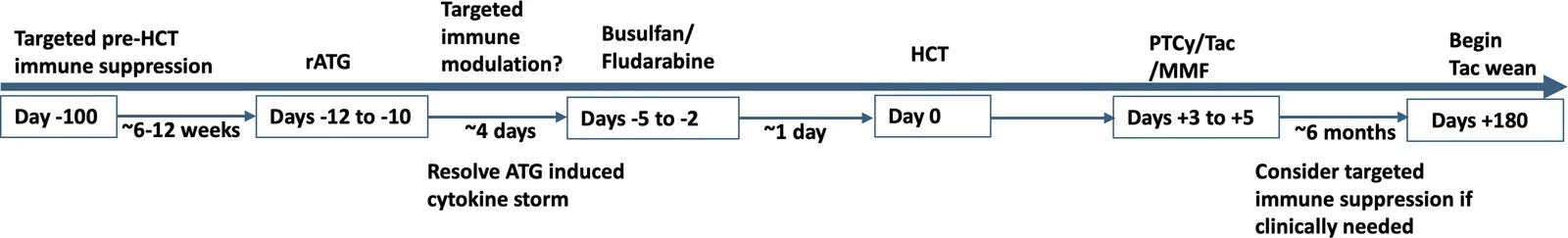
**HCT preparatory regimen template.** A typical conditioning regimen based on our approach to HCT for patients with PIRDs and/or inflammatory disorders. A block of targeted PTIS for 6–12 wk to bridge patients to HCT should be incorporated based on IDDA2.1 scores and clinical phenotypes. This is followed by model-based ATG, busulfan, and fludarabine +/− additional immune suppression if needed. GVHD prophylaxis is accomplished with PTCy, tacrolimus (Tac), and MMF. Consideration of additional posttransplant targeted immune modulation based on the clinical phenotype of the patient.

The question of what level of donor myeloid (and/or T cell) chimerism is sufficient to ameliorate disease activity is one that is constantly debated and likely different across patients with various PIRDs, autoinflammatory, and/or hyperinflammatory syndromes. For this reason, our approach has been to aim for full donor myeloid chimerism whenever possible. We therefore use a standard myeloablative, reduced-toxicity backbone of busulfan and fludarabine as our myeloablative and immune-ablative agents (Fig. 3). For busulfan, we use real-time PK-adjusted dosing to target a cumulative area under the curve (cAUC) of 80–90 mg*hr/L (92, 93). Other centers take an age-based approach (i.e., inversely correlating busulfan cAUC targets with age) and/or stratify busulfan cAUC targets based on disease (e.g., higher busulfan cAUC targets for GOF and lower targets for loss-of-function disorders). Given the rarity of each of these syndromes, there is not enough data to adjudicate the optimal approach. For fludarabine, we use a population-PK model to target a cAUC of 20 mg*hr/L (94). When possible, we attempt to administer the busulfan and fludarabine as close to day 0 as possible (i.e., days −5 to −2) so that the patient is at nadir for the shortest possible time. This regimen of rATG/busulfan/fludarabine has a lot of flexibility, and we previously published on how we interlaced emapalumab into the regimen for a patient with STAT1 GOF (42). We have also included other targeted agents (e.g., MAS825) in this conditioning framework for other diseases. The tight control of busulfan exposure allows us to reduce intensity for patients with end-organ (e.g., lung or liver) toxicity. For instance, a patient with borderline pulmonary function tests may receive this regimen with a targeted busulfan cAUC of 60 mg*hr/L to try and avoid additional lung strain. Thus, having one standard regimen has allowed us to gain deep experience with the patient journey but also allows flexibility to personalize and optimize the regimen for each individual patient.

Though we attempt to optimize our conditioning regimen to target full donor myeloid (and T cell) chimerism, mixed donor myeloid and/or T cell chimerism may still occur. For mixed or falling donor myeloid chimerism, we would typically monitor the patient closely and potentially consider a CD34^+^ selected boost if there were concurrent cytopenias. If donor T cell chimerism is mixed or falling, our institutional approach has been to avoid donor lymphocyte infusions due to the risk of GVHD. Instead, we monitor closely and, if the graft fails, pursue a second transplant if necessary. These approaches though, require additional prospective study and data to evaluate optimal strategies.

Many other regimens exist, and though they may not be the standard at our center, there is limited, if any, data to adjudicate which approach is best. Despite being an excellent immune ablative and/or myeloablative agent (depending on total delivered dose), we do not use any total body irradiation for this patient population, as the long-term side effects likely outweigh potential benefit in most patients. The recent approval of treosulfan in the United States has provided a very interesting agent, particularly for patients with pre-existing lung or liver toxicity. Treosulfan is a myeloablative agent; however, recent data in patients with Wiskott–Aldrich syndrome suggests that a treosulfan/fludarabine +/− thiotepa regimen had a graft failure rate of 14.3% compared with 2.9% in the busulfan/fludarabine +/− thiotepa-treated patients (95). Whether this was because the treosulfan exposure was not optimized for patients or because treosulfan is not as good a myeloablative agent as busulfan needs to be determined. It will be interesting to evaluate treosulfan as additional data on exposure-based dosing and PK models are developed and trialed.

Some centers prefer a reduced-intensity approach based on a melphalan and fludarabine backbone (96). Though there are different iterations of this regimen, the most common is alemtuzumab, fludarabine, melphalan, and thiotepa (97). The benefit of this approach is the avoidance of busulfan and related potential toxicities, especially in patients coming to alloHCT with end-organ damage. The drawbacks, however, include higher rates of mixed donor chimerism, graft rejection, and need for CD34^+^ selected boosts and/or second alloHCT (58, 96, 98). Similar to our data using emapalumab with rATG, busulfan, and fludarabine, data using emapalumab with the alemtuzumab, fludarabine, melphalan, and thiotepa regimen in patients with pHLH showed improved donor chimerism (43). Whether using reduced-intensity conditioning, knowing that many patients will do well but a subset will require further intervention, is superior or inferior to a myeloablative approach with busulfan is not known, as prospective clinical trials to compare these regimens have not yet been done, but it is a question that should be prospectively explored.

## GVHD prophylaxis

Advances in supportive care and GVHD prophylaxis have enabled the safe use of alternative donors and significantly expanded the available donor pool. The two major strategies rely on either ex vivo graft manipulation (TCRαβ T cell depletion) or in vivo T cell depletion with PTCy (99). PTCy works by eliminating activated (alloreactive) T cells on days +3 and +4 (100). Aside from deep GVHD prophylaxis, PTCy can also remove residual activated host T cells that may not have been sufficiently depleted during conditioning. Thus, we can provide GVHD prophylaxis while allowing for one last immune ablation of host T cells to minimize graft rejection risk. We have therefore made PTCy, tacrolimus, and MMF our standard GVHD prophylaxis regimen irrespective of donor choice in the PIRD, autoinflammation, and hyperinflammation setting. Our standard approach for PIRD and/or inflammatory disorders is therefore rATG, busulfan, and fludarabine with PTCy, tacrolimus, and MMF, which can be individualized as previously described.

An alternative approach to GVHD prophylaxis involves ex vivo graft manipulation either through CD34^+^ selection or TCRαβ T cell depletion of mobilized peripheral stem cells (101, 102). The benefits of this approach include delivery of high CD34^+^ stem cell doses, shorter time to engraftment, faster discharge home, and not exposing new hematopoietic stem cells of the graft to alkylating chemotherapy. The downsides include the expense of graft engineering, delivery of a stem cell product without the full complement of marrow niche cells, and higher reported rates of graft rejection. In a recent retrospective multicenter study comparing PTCy with TCRαβ T cell depletion for alternative donor alloHCT in patients with IEIs (inclusive of many non-PIRD disorders), PTCy was associated with increased severe acute GVHD, veno-occlusive disease, acute kidney injury, and pulmonary complications, while TCRαβ T cell depletion had higher rates of adenoviremia, graft failure, and increased risk of second alloHCT (99). The conditioning approaches varied significantly within each group, and thus, the authors conclude that either platform is appropriate for alloHCT in patients with IEI and should be further tested in a prospective clinical trial.

## Posttransplant immune suppression

One of the most complicated factors of alloHCT for patients with PIRDs and/or inflammatory disorders is deciding when additional immune suppression should be introduced after alloHCT (in addition to standard GVHD prophylaxis). This concept is difficult because traditional dogma is that rapid immune reconstitution is essential for improved alloHCT outcomes and decreased opportunistic infections. The association between immune (specifically CD4^+^ T cell) reconstitution and decreased transplant-related morbidity has previously been shown (84). In patients receiving alloHCT for hematologic malignancies, robust immune reconstitution is likely helpful for the graft-versus-leukemia effect. Thus, increasing immune suppression after alloHCT runs counter to what we aim to accomplish for most other patients. Patients with PIRDs, autoinflammation, or hyperinflammation, however, present a unique set of issues, intrinsic to their underlying diagnosis, that makes the consideration of additional immune suppression necessary. First, tissue-resident immune cells can take months to years to transition from host- to donor-derived, and until a critical number of these cells convert to donor, they still harbor the potential for dysregulated immunity (103). Second, many of these disorders affect both hematopoietic and extra-hematopoietic cells (20). As alloHCT only replaces the hematopoietic compartment, extra-hematopoietic manifestations of disease can still occur.

### Case 2 continued

Since additional immune suppression presents significant risk, we allow each patient to declare if they require intervention. Returning to our patient with *IKZF1* GOF who received PTIS with ruxolitinib, with improvement in lymphadenopathy, splenomegaly, and inflammatory lung disease. Ruxolitinib was tapered off, followed by MUD alloHCT conditioned with emapalumab, rATG, busulfan (cAUC = 90 mg*hr/L), fludarabine (cAUC = 20 mg*hr/L), and PTCy, tacrolimus, and MMF for GVHD prophylaxis. Engraftment was slow but did occur. Letermovir, tacrolimus, and MMF were discontinued on day +28 when a second alloHCT was considered, but the patient ultimately engrafted and was discharged home off immune suppression. He did well for several weeks, though he did have low-level CMV reactivation, but then got readmitted for a sepsis rule-out in the setting of fevers and began to have an oxygen requirement. Donor chimerism was >95% at that time. Comprehensive sepsis assessment was negative. The central line was pulled without improvement. Imaging of the lungs showed innumerable ground-glass opacities. Bronchoalveolar lavage and biopsy did not yield an infectious etiology. Pulmonary function tests were significantly compromised compared with prior readings. Throughout this episode, CMV viremia began to slowly rise. Our group had a heated debate about whether to continue pursuing anti-infectious therapies or to initiate immune suppression, especially given the rising CMV viremia. The patient was ultimately started on methylprednisolone with rapid resolution of fever and oxygen requirement. Vital signs normalized, and CMV viremia began to improve despite being on steroids, indicating that this was likely an immune dysregulation that needed treatment. Tacrolimus was added, and the patient ultimately had a slow steroid taper with complete resolution of lung pathology to allow for continued conversion of tissue-resident immune cells.

It is therefore critical to consider organ-specific immune dysregulation in the post-alloHCT setting. First and foremost, common post-alloHCT complications such as sepsis, opportunistic infection, GVHD, and endothelial damage must be ruled out. If, however, clinical symptoms or workup are not consistent with these pathologies, a high degree of suspicion should be maintained for residual immune dysregulation, especially if the target organ or symptom pattern is similar to pre-alloHCT pathology. In our experience, ∼30% of patients required some additional immune suppression to manage post-alloHCT immune dysregulation. In most cases, this was organ-specific and not systemic immune dysregulation. Most patients were able to come off immune suppression after slow tapers to allow for sufficient conversion of tissue-resident immune cells. In rare cases, low-dose or intermittent targeted immune suppression may be required to manage extra-hematopoietic manifestations of the underlying disorder. These posttransplant immune and inflammatory complications are still being explored. Predictive biomarkers, prospective studies, and consensus definitions are needed as we continue to learn about these unique phenomena.

## Long-term monitoring

Similar to pre-alloHCT, a multidisciplinary long-term follow-up team provides optimal care for this patient population. Aside from traditional long-term alloHCT and chemotherapy follow-up, additional surveillance is required. While certain pathologies such as inflammatory lung disease and inflammatory bowel disease usually (though not always) improve or resolve after alloHCT (104, 105), other pathologies may not resolve and need close monitoring by appropriate sub-specialists. An immunologist, rheumatologist, and/or physician who specializes in immune dysregulation and hyperinflammatory syndromes should be involved. In addition, some of these conditions predispose to a variety of long-term risks for which a surveillance program should be established. Genetic counseling and family planning should be offered when appropriate. Finally, immune function and disease resolution should be measured by clinical laboratory assessments and longitudinal objective scoring (e.g., IDDA2.1 scores) to delineate the benefit we are providing and identify areas for improvement (67, 106).

## Future directions

The field of alloHCT for PIRDs and inflammatory disorders has come a long way. We are now able to provide cures with improved outcomes for tough-to-treat disorders like STAT1 GOF. Despite improved outcomes, there are still many areas for refinement to minimize toxicity and continue to improve OS, EFS, and patient quality of life. Most importantly, identifying patients appropriate for curative therapy early in their disease process remains challenging, and continued work in this area is needed. Validating predictive biomarkers that portend disease progression or severity of disease would be invaluable to provide additional objective data to assist providers and families in making informed decisions about proceeding or not proceeding with alloHCT. As the field is relatively new, continued partnership with immunologists, especially at centers that might not have a PIRD and/or hyperinflammation multidisciplinary team, is crucial to make sure that patients do not miss a potential window for curative therapy. At our center, all patients with PIRD or hyperinflammatory syndromes are seen at least one time by a member of the transplant team so that families and physicians are aware of potential curative options. Additional opportunities for collaboration and trainee education amongst pediatric and adult providers in immunology, rheumatology, hematology/oncology, and HCT are critical to continue moving the field forward.

Exposure and model-based conditioning regimens promote more precise delivery of myeloablative and immune ablative drugs (92, 93, 94). We are still, however, reliant on alkylating agents and the side effects and sequelae thereof. There have been advances in antibody-based conditioning regimens, though not yet ready for clinical implementation (107, 108, 109, 110). Anti-CD117 monoclonal antibodies have been used in clinical trials of alloHCT for malignancy (111), SCID (NCT02963064), GATA2 haploinsufficiency (NCT05907746), chronic granulomatous disease (NCT05600907), Fanconi anemia (112) (NCT04784052), and sickle cell disease (NCT05357482) with varying success. Anti-CD45 antibody conjugated to I-131 has been studied in the alloHCT setting for malignancy (113) and is currently being studied in alloHCT for sickle cell disease (NCT07015684). While there have been setbacks in the development of antibody-based conditioning, continued refinement of antibodies and antibody-drug conjugates for conditioning are critical to minimize or eliminate the use of alkylators and continue to improve patient outcomes (108, 114, 115).

While alloHCT is the current standard of care for curative approaches in this patient population, several studies in autologous gene editing and gene therapy strategies are beginning to emerge. Waskyra (etuvetidigene-autotemcel), an ex vivo lentiviral-based gene therapy for Wiskott–Aldrich syndrome (116), was recently approved by the Food and Drug Administration. There is an ongoing lentiviral gene therapy of patient-derived CD4^+^ T cells in IPEX syndrome (NCT05241444) (117). Preclinical data in gene therapy and gene editing strategies are emerging in pHLH syndromes (118, 119, 120), STAT1 GOF, and others. The benefits of these therapies are that they use patient-derived cells or are even infused in vivo without the need for ex vivo manipulation. The first such in vivo gene therapy clinical trial for IEI is currently accruing patients with X-linked chronic granulomatous disease and does not require the use of classical conditioning agents, though it does use low-dose chemotherapy as part of subsequent enrichment cycles (NCT06876363). The amount of correction these therapies can provide is yet to be determined. How to scale up and make these bespoke drug products affordable and viable for greater patient access is also a topic that has engendered much discussion. These therapies may also be useful for patients who are ineligible for alloHCT because they missed their window or don’t have appropriate available donors.

Major advances have thus been made in the field of curative therapies for PIRDs and/or inflammatory disorders. OS and EFS have improved across the board, and the use of alternative donors now allows us to deliver cures to many more patients. Bridging patients with targeted immune modulation and PTIS improves their fitness for alloHCT and ultimately their outcomes. Distinct posttransplant complications may occur from the underlying disease and need to be quickly identified and addressed. Partnerships between pediatric and adult providers will continue to expand the number of patients that can be considered for cure. As the field continues to evolve, it is critical for patients to be identified early, referred to a program with multidisciplinary expertise and for patients to be closely followed so that if curative therapy is right for them, it can be done at the optimal time.
